# Sexual Fantasies and Stereotypical Gender Roles: The Influence of Sexual Orientation, Gender and Social Pressure in a Sample of Italian Young-Adults

**DOI:** 10.3389/fpsyg.2019.02864

**Published:** 2020-01-15

**Authors:** Carla Tortora, Giulio D’Urso, Filippo M. Nimbi, Ugo Pace, Daniela Marchetti, Lilybeth Fontanesi

**Affiliations:** ^1^Department of General Psychology, University of Padua, Padua, Italy; ^2^Faculty of Human and Society Sciences, Kore University of Enna, Enna, Italy; ^3^Department of Dynamic and Clinical Psychology, Sapienza University of Rome, Rome, Italy; ^4^Department of Psychological, Health and Territorial Sciences, G. d’Annunzio University of Chieti-Pescara, Chieti, Italy

**Keywords:** sexual fantasies, LG, gender role, stereotypes, social pressure

## Abstract

Differences in gender and sexual orientation are suggested to be linked to differences in the way individuals think and behave. The aim of the current study is to evaluate the effect of gender and sexual orientation on sexual fantasies and gender roles in heterosexual and gay and lesbian people. The sample was composed of 547 participants, 246 men (*M*_age_ = 28.85; SD = 9,27) and 301 women (*M*_age_ = 25,97; SD = 7,141). Within this sample, 61.8% of men and 79.4% of women were heterosexual, whereas 38.2% of men and 20.6% of women were gay and lesbian. Participants completed an online battery of questionnaires to assess their sexual orientation, sexual fantasies, and gender roles on three different dimensions. It was hypothesized that the heterosexual group would report more normative sexual fantasies (H1) and that women in general would report androgynous characteristics, which would be linked to a low degree of reported feminine ideal roles and high social pressure to conform to feminine social expectations (H2). The results showed that lesbian women scored slightly higher than heterosexual women on transgressive sexual fantasies and lower on emotional-romantic ones. Moreover, heterosexual women, but not lesbian women, showed a pattern of high social pressure to conform to feminine expectations together with lower scores in the IRQ. We found the same results on gay men but not for heterosexual men. The overall results suggest that sexual fantasies and gender roles are relatively independent concepts and are influenced by different mechanisms.

## Introduction

The term sexual fantasy refers to sexually arousing mental images (Leitenberg and Henning, 1995) that have a drive-facilitating role (Knafo and Jaffe, 1984) and that can actually enhance sexual experience (Sue, 1979). Hence, sexual fantasies, even those considered “deviant,” are a component of healthy sexual life (Sue, 1979), deemed to be common to everyone (Brown and Hart, 1977; Price and Miller, 1984); some have even defined them as a type of cognitive skill (Knafo and Jaffe, 1984). Sexual fantasies play a major role in influencing later sexual behavior and in reflecting past experiences and are a core variable in the systematic study of sexual identity and sexuality (Fontanesi and Renaud, 2014). The erotic imaginary can both influence and be influenced by personal experiences, but a consistent branch of research suggests that sexual fantasies are a strong and clear example of the differences in the development of male and female sexuality in our species (Wilson, 1997; Yost and Zurbriggen, 2006). According to this idea, the current study focuses on the hypothesis that gender, sexual orientation, and gender roles can influence the direction of sexual fantasies in a more feminine or masculine direction and, consequently, sexual behavior and sexual wellbeing.

### Sexual Fantasies and Gender Differences

Some research has focused on studying and assessing the personality traits associated with specific sexual fantasy themes, yet there is little agreement on the factors according to which it is possible to categorize sexual fantasies. These conflicting results are a consequence, at least in part, of the lack of a common sample of fantasy themes that can be used to assess them (Moyano and Sierra, 2013). Nonetheless, sexual fantasizing seems to be negatively correlated with anxiety (Birnbaum et al., 2012) and positively related to self-esteem and security (Renaud and Byers, 2001). Since the trait anxiety is typically associated with women, it is not surprising that sex differences have been intensively explored. Sex guilt, which is also more present in women than men, is one factor that has been found to be an important modulator of the quantity, embarrassment, vividness, explicitness, and variety of reported sexual fantasies, as well as time and morals, which tend to influence the willingness to report sexual fantasies (Wolke et al., 2000; Fontanesi and Renaud, 2014; Goldey et al., 2014; Panzeri and Fontanesi, 2014). Furthermore, it has been suggested that men have more fantasies than women (Baumeister et al., 2001), although, during intercourse, this does not seem to be the case (Leitenberg and Henning, 1995). It is important to stress that the quantity of sexual fantasies in women increases with age (Brown and Hart, 1977; Pelletier and Herold, 1988; Panzeri and Fontanesi, 2014) and that, interestingly, differences in incidence were only found during masturbation, while they were no longer significantly present during intercourse and sexual daydreaming (Leitenberg and Henning, 1995). Nevertheless, according to the literature, the kinds of themes of sexual fantasies seems to vary significantly between men and women (Zurbriggen and Yost, 2004; Carpenter et al., 2008), with men reporting more sexual themes than women (Leitenberg and Henning, 1995). In general, explicitness has been linked to men, while emotional-romantic contents, to women (Yost and Zurbriggen, 2006), although these results have not always been replicated and might now be outdated, at least for some young populations (Allen, 2003). Indeed, the latter is supported by findings that more similarities than differences may be present in both the frequency and content of sexual fantasizing (Cado and Leitenberg, 1990). Some differences have also been found in the purposes of fantasies: women may emphasize their own needs more (Zurbriggen and Yost, 2004) and may use the fantasies to deviate from traditional scripts (Goldey et al., 2014), maybe due to their traditionally having a more passive role in sex as well as in social life. Sociobiological and sociocultural theories have been offered to account for gender differences in sexual behavior and attitudes, including sexual fantasies. In both models, it is generally predicted that women will be more cautious than men in partner selection and in having sexual activity outside of a relationship (Singer, 1985). Despite some contradictory results regarding differences between heterosexual and gay and lesbian individuals, it emerges that males share the same pattern of preferences regardless of their sexual orientation and of cultural differences (Masters et al., 1982; Price et al., 1985; Bhugra et al., 2006).

### Culture and Sexual Fantasies

Sexual fantasies in women were initially thought to be comparable across different ethnicities (Shanor, 1977), yet it has been proposed that this may not be the case (Pietropinto and Simenauer, 1977; Price and Miller, 1984). It was accordingly stated that differences might be the result of variation in willingness to report fantasies that are considered less acceptable by the society (Critelli and Bivona, 2008). Indeed, more recent research has found culture to play an important role in the expression of sexuality (Traeen et al., 2002), and this has become particularly evident when comparing European countries with, for instance, some Asian countries, in which sex differences in social roles, occupations, and expectations are amplified (Usui et al., 2003; Yamamoto and Ran, 2014; Smirles, 2017). The latter, indeed, show a general pattern of conservative attitudes about sexuality as well as limited knowledge about sexuality, regardless of biological sex (Okazaki, 2002; Nobre et al., 2003). These results, however, may apply more consistently to heterosexual men than gay men (Bhugra et al., 2006). The role of culture is not surprising since it modulates the emotions and behaviors of the group (Tseng, 2003). Notably, culture is considered to be largely learned, not innate (Heine et al., 1999). If these assumptions are true, very different cultures should give rise to very different representations of and expectations about sexuality and, consequently, sexual fantasies as well.

### Gender Roles and Stereotypes

Since gender is suggested to be a key factor in shaping many domains, it has been described as an important predictor of sexual behaviors, sexual attitudes, and romantic relationships (Wu et al., 2016). Gender is not a unidimensional concept; it arises from self-representations that comprise the psychological, behavioral, and social consequences of self-perceived gender (American Psychiatric Association, 2013). While sex is biologically determined, gender is a socially constructed concept that is developed through experience with the social context (Bem, 1993; Martin and Ruble, 2010; Fausto-Sterling, 2012). Accordingly, stereotypes about gender are not fixed, and they can be successfully modified (Nathanson et al., 2006). Particularly, gender is linked to “the widely shared set of expectations and norms linked to how girls and boys should behave” (Kabbash et al., 2019, p. 331), and, thus, to specific tasks, personalities, and behaviors that individuals are “expected” to embrace accordingly (Littlefield, 2004; Nguyen et al., 2010; Hall and Pichon, 2014). However, identifying oneself with a gender does not necessarily imply endorsing its stereotypical roles (Kite et al., 2008; Dean and Tate, 2017). This appears to be especially true for women, whose gender stereotypes tend to be more dynamic in time (Diekman and Eagly, 2000; Koch et al., 2005; Zafra and Garcia-Retamero, 2011; Diekman et al., 2013; Bosak et al., 2017) and across countries (Weinberg et al., 2000; Higgins et al., 2002; Zafra and Garcia-Retamero, 2011; Constantin and Voicu, 2014; Hall and Pichon, 2014).

It was also proposed that sex plays a much more important role than culture in shaping gender roles (D’Urso and Pace, 2019), probably due to the generally accepted assumption that women are warm, sensitive, and caring, while men are assertive, dominant, and competitive (Usui et al., 2003; Bosak et al., 2017). Nevertheless, these appear to be biased attributions that may rather be the result of stereotypes and schemas acquired in early childhood and maintained through mechanisms such as social rewards or punishments (Bandura, 1986), correspondence bias (Ross, 1977), behavioral confirmation (Geis, 1993; Kite et al., 2008), and stereotype threat (Steele, 1997), in line with social role theory (Eagly, 1987; Diekman and Eagly, 2000; Kite et al., 2008) and social learning theory (Gelman et al., 2004; D’Urso et al., 2019). Furthermore, it has been noted that boys tend to receive much more criticism when typically feminine characteristics are acted out (Martin and Fabes, 2001; Martin and Ruble, 2010). As a consequence, while a consistent shift in the traits commonly associated with women has occurred in the last few decades, the acquisition of female attributions by men is less substantial (Diekman and Eagly, 2000; Zafra and Garcia-Retamero, 2011). Indeed, gender stereotypes can be used to assess the degree of sex differences and sexual orientation in a society (Eagly, 1987; Zafra and Garcia-Retamero, 2011). Many people, especially those with little or no contact with gay men and lesbian women (Salvati et al., 2019), may believe that a direct link exists between sexual orientation and gender roles (Lehavot and Lambert, 2007). Two main mechanisms are responsible for this biased association: firstly, people tend to assume that gay men and lesbian women hold counter-stereotypical gender roles (Sirin et al., 2004; Blashill and Powlishta, 2009); and secondly, individuals who deviate from their stereotypical roles may be assumed to have an homosexual orientation, regardless of their actual sexual orientation (Sirin et al., 2004; Cox et al., 2016). As a result, gay men may experience pressure to adhere to stereotypically masculine roles, traits, and behaviors due to perceived threats to their masculinity (Hunt et al., 2015). Furthermore gay men and lesbian women may react negatively to feminine gay men (Salvati et al., 2017). For the aforementioned reasons, the social context as well as the individual strains, if present, to counteract stereotypes associated with being a gay man or a lesbian woman may have an influence on results when assessing gender roles in gay men and lesbian women. Notably, gaining new counter-stereotypical traits does not imply losing the stereotypical ones (Bosak et al., 2017), suggesting a possible shift toward androgyny; that is, masculine and feminine traits being equally present in the same individual (Bem, 1974).

Finally, when describing gender and gender roles, it is essential to take into account that gender roles are schemas of the self. Edward Tory Higgins, in *Self-Discrepancy Theory*, described the self as characterized by three domains: actual (who the person is), ideal (how the person would like to be), and ought (how the person is expected to be by the society) (Higgins, 1987). Therefore, it may be reasonable to assume that gender and gender roles hold the same domains. If this is true, then discrepancies related to these three dimensions may occur, leading to negative outcomes for the person (Higgins, 1987). However, androgynous traits provide individuals with the flexibility to adapt to different settings and to operate differently according to their needs (Bem, 1974). Accordingly, discrepancy is expected to produce negative effects only in strongly sex-typed individuals.

### The Current Study

The aim of the current study is to assess the influence of gender and sexual orientation on sexual fantasies and gender roles in Italian heterosexual and gay and lesbian samples. The intention is not to limit our understanding to the exploration of inter-group differences but rather to provide some insights also into intra-group, individual differences, especially regarding the dimensions according to which gender can be classified. Specifically, we hypothesized that heterosexual individuals would report more normative sexual fantasies than their gay and lesbian counterparts (H1) (Bhugra et al., 2006). Furthermore, a shift toward androgynous characteristics was expected in women (Diekman and Eagly, 2000; Zafra and Garcia-Retamero, 2011), which was predicted to be linked to a pattern of a low intensity of feminine ideal roles and high social pressure to conform to feminine social expectations (H2).

## Materials and Methods

### Participants and Procedure

Participants were randomly recruited online through public groups on social networks (e.g., Facebook) and participated in the research on a voluntary basis. Data were collected by the authors using “Google Forms” through an online battery of questionnaires that took about 20 min to complete. The survey was spread from March to July 2019. Inclusion criteria were being at least 18 years old, being Italian, and understanding the Italian language; exclusion criteria for the present study were being non-binary, bisexual, or asexual. The research was conducted in accordance with the ethical principles stated in the Declaration of Helsinki (2013).

The initial sample was composed of 625 participants. In total, 78 participants were excluded from the data analysis: 41 of them reported a non-binary gender, 33 of them being bisexual and the other four asexual, which were not topics in the present investigation. Therefore, the final sample was composed of 547 participants, 246 men (*M*_age_ = 28.85; SD = 9,27) and 301 women (*M*_age_ = 25,97; SD = 7,141). According to Kinsey’s scale (Kinsey’s et al., 1948), 61.8% of the men and 79.4% of the women were heterosexual; 38.2% of the men and 20.6% of the women were lesbian. Students made up 53.9% of the sample. The age ranged from 18 to 66 (*M* = 27.24; SD = 8.29). Most of the participants (77.3%) were between 18 and 30 years old. The overall educational level was relatively high: 45.9% of participants had graduated from high school, 27.2% of them had a bachelor’s degree, and 14.1% of them had a master’s degree. In terms of relationship status, 38.9% of the sample was single, 48.6% were in a relationship, while the remaining 12.4% were married or separated. Only 4.4% of the participants had at least one child. Finally, 73.7% of the sample were atheists or agnostic.

### Measures

The online battery included socio-demographic questions about biological sex, self-perceived gender, age, sexual orientation, nationality, age, educational level, civil status, number of children, employment situation, and religiosity.

#### Heterosexual-Homosexual Rating Scale

The Heterosexual-Homosexual Rating Scale (Kinsey’s et al., 1948) was used to assess the sexual orientation of each participant on a continuum that ranged from 0 (“Exclusively heterosexual”) to 6 (“Exclusively homosexual”) and that included asexuality. Specifically, values from 0 to 2 were considered indicators of heterosexuality; values from 4 to 6 were assigned to homosexuality; the value 3 corresponded to bisexuality; finally, the letter “*x*” represented asexuality.

#### Erotic Imagery Questionnaire

The Erotic Imagery Questionnaire (EIQ) (Panzeri et al., 2015) is an Italian questionnaire that appraises sexual fantasies based on five major factors: transgressive theme (α = 0.77) (F1; e.g., “Do you fantasize about role-playing with your partner?”), emotional-romantic theme (α = 0.63) (F2; e.g., “In your sexual fantasies, do you fantasize about a love story that ends with sexual intercourse?”), dominance/submission theme (α = 0.74) (F3; e.g., “Do you ever fantasize about situations in which you are forced to have sex?”), variety of partners (α = 0.70) (F4; e.g., “Would you say that fantasizing about having sex with someone you hired for a job, such as a plumber, a gardener, or a maid, is part of your erotic imagery?”), and sexually explicit images (α = 0.68) (F5; e.g., “In your sexual fantasies, is it important for you to visually focus on your partner’s genitals?”). Participants were asked to rate the extent to which the content of the 41 items applies to them on a five-point scale ranging from 1 (“Never”) to 5 (“Always”).

#### Bem Sex-Role Inventory

The Bem Sex-Role Inventory (BSRI) (Bem, 1974) evaluates the degree to which respondents adhere to stereotypical gender traits (Bem, 1974), which were found to be strictly linked to the endorsement of implicit gender stereotypes at large, including roles (Rudman and Phelan, 2010); thus, the BSRI was considered indicative of the “actual” gender roles of each participant, as defined in the previous introduction. Additionally, despite being often criticized (Hoffman and Borders, 2001), it is still the most used instrument for assessing gender stereotypes (Hoffman and Borders, 2001; Starr and Zurbriggen, 2017), thus allowing an evaluation of how attitudes about gender have changed over time (Lips, 2017). The BSRI is a structured questionnaire composed of a total of 60 items, 20 of which indicate neutral personality characteristics (α = 0.76), 20 masculine (α = 0.89), and 20 feminine (α = 0.85). Respondents were asked to indicate the extent to which each personality characteristic applies to them on a seven-point scale ranging from 1 (“Never or almost never true”) to 7 (“Always or almost always true”) (Bem, 1974).

#### Ideal Roles Questionnaire and Stereotype Pressure Questionnaire

Due to the lack of validated Italian instruments for assessing the “ideal” roles as well as the social pressure to conform to the stereotypical ones, two *ad hoc* structured questionnaires were developed on the basis of the existing literature about social stereotypes (Bem, 1974; Usui et al., 2003; Bosak et al., 2017). The Ideal Roles Questionnaire (IRQ) comprehended 28 items (α = 0.84) representing roles and behaviors typically associated with women (e.g., “I would like to leave important decisions to my partner”). This choice was due to the desire to assess whether the hypothesis that women tend to deviate from their stereotypical roles more than men (Diekman and Eagly, 2000; Zafra and Garcia-Retamero, 2011) could be expanded also to the “desire” sphere or whether it is rather limited to the “actual” one due to social pressure or internalized stereotypes. Participants rated on a seven-point scale the degree to which they would like to adopt a certain role or behavior ranging from 1 (“I completely disagree”) to 7 (“I completely agree”). Since both the IRQ and the BSRI were supposed to be indicators of gender roles and traits, the construct validity of the IRQ was evaluated by testing the hypothesis that sex-typed individuals should report stereotypical roles and traits coherently on both the measuring instruments. A strong positive correlation was found between the IRQ and the feminine subscale of the BSRI (*r*_*s*_ = 0.58; *p* < 0.01), while the IRQ and the masculine subscale of the BSRI were strongly negatively correlated (*r*_*s*_ = −0.53; *p* < 0.01). Moreover, an explorative factors analysis identified one principal factor explaining 45.5% of variance, which has been considered sufficient, in relation to other measures indicated, to assess the ideal role.

The Stereotype Pressure Questionnaire (SPQ) included 28 items divided into two subscales: 15 of them (α = 0.86) referred to the social pressure to conform to masculine characteristics (e.g., “being the main source of income in the family”), while the other 13 (α = 0.84) to the feminine ones (e.g., “working less in order to devote more time to the family”). Participants were asked whether they experienced pressure to adopt a role or behavior on a seven-point scale ranging from 1 (“Never”) to 7 (“Always”). The questionnaire was constructed starting from the IRQ, the construct validity of which in the differentiation between feminine and masculine roles was verified.

### Analysis Plan

Statistical analyses on the sample were performed using SPSS software (IBM, 2012). ANOVA and MANOVA analyses were conducted to assess the influence of gender and sexual orientation as well as the interaction of the two with the EIQ factors. Before the analyses were performed, linearity, homoscedasticity, normality of residuals, and multicollinearity assumptions were assessed. Data were not organized on a normal distribution, but the deviation from normality was minimal, with both asymmetry and skewness around 0. Thus, statistical analyses were computed.

## Results

Table 1 shows the Pearson correlation between the study variables and total scores. All of the EIQ factors positively correlated with both feminine and masculine gender roles, and significant positive correlation was found between IRQ and the feminine subscale of the SPQ (*r* = 0.332; *p* < 0.01). The second table (Table 2) reports statistical descriptions (means and standard deviations) for gender and sexual orientation. The IRQ scores are equivalent for what concerns the samples of men and women, but men scored higher than women on the transgressive theme (F1), variety of partners (F4), and sexually explicit images (F5) of the EIQ, while women scored higher on the emotional-romantic theme (F2) and dominance/submission theme (F3) as well as on the feminine subscale of the SPQ and on the feminine subscale of the BSRI.

**TABLE 1:** Pearson correlations between the study variables and total scores (mean, ds, asymmetry, and kurtosis).

**Variable**	**1**	**2**	**3**	**4**	**5**	**6**	**7**	**8**	**9**	**10**	**11**
1. F1^1^	–										
2. F2^2^	0.339^∗∗^	–									
3. F3^3^	0.514^∗∗^	0.130^∗∗^	–								
4. F4^4^	0.436^∗∗^	0.140^∗∗^	0.357^∗∗^	–							
5. F5^5^	0.426^∗∗^	0.156^∗∗^	0.267^∗∗^	0.516^∗∗^	–						
6. IRQ	0.092^∗^	0.205^∗∗^	0.003	0.059	0.162^∗∗^	–					
7. SPQ_M	0.201^∗∗^	0.171^∗∗^	0.169^∗∗^	0.222^∗∗^	0.148^∗∗^	0.315^∗∗^	–				
8. SPQ_F	0.209^∗∗^	0.187^∗∗^	0.216^∗∗^	0.196^∗∗^	0.131^∗∗^	0.332^∗∗^	0.738^∗∗^	–			
9. BSRI_M	0.226^∗∗^	0.094^∗^	0.155^∗∗^	0.165^∗∗^	0.095^∗^	–0.153^∗∗^	0.207^∗∗^	0.149^∗∗^	–		
10. BSRI_F	0.099^∗^	0.269^∗∗^	0.097^∗^	0.024	0.055	0.334^∗∗^	0.121^∗∗^	0.183^∗∗^	0.352^∗∗^	–	
11. BSRI_N	0.187^∗∗^	0.216^∗∗^	0.112^∗∗^	0.124^∗∗^	0.103^∗∗^	0.266^∗∗^	0.234^∗∗^	0.228^∗∗^	0.542^∗∗^	0.790^∗∗^	–
M	23.94	25.36	18.50	13.52	10.65	118.54	47.92	46.40	4.24	4.48	4.31
SD	6.97	5.53	6.07	4.44	3.87	20.89	14.95	14.39	0.93	0.76	0.62
Asymmetry	0.15	–0.12	0.42	0.49	0.65	–0.10	0.28	0.03	–0.05	–0.27	–0.29
Curtosis	–0.44	–0.39	–0.44	–0.16	–0.04	0.74	0.04	–0.22	–0.18	0.60	1.1

*^∗∗^*p* < 0.01, ^∗^*p* < 0.05. EIQ factors: ^1^transgressive theme; ^2^emotional and romantic theme; ^3^dominance/submission; ^4^variety of partners; ^5^explicit sexuality.*

**TABLE 2:** Means and standard deviations for the study variables, divided by gender and sexual orientation.

	**Males (*N* = 246)**		**Females (*N* = 301)**		**Heterosexuals (*N* = 391)**		**Gay men and lesbian women (*N* = 156)**	
**Variable**	***M***	**SD**	***M***	**SD**	***M***	**SD**	***M***	**SD**
**EIQ**								
F1^1^	25.15	6.88	22.94	6.89	24.03	6.88	23.70	7.19
F2^2^	24.22	5.26	26.29	5.58	26.04	5.44	23.66	5.40
F3^3^	17.67	5.77	19.18	6.24	18.08	6.23	19.54	5.55
F4^4^	14.93	4.60	12.36	3.95	12.91	3.98	15.02	5.13
F5^5^	11.76	4.15	9.73	3.37	10.05	3.43	12.15	4.46
IRQ	118.80	21.44	118.33	20.47	117.04	20.75	122.09	20.84
SPQ_M	47.08	15.84	48.61	14.16	47.46	14.36	49.09	16.30
SPQ_F	44.39	14.11	48.04	14.43	45.83	14.21	47.82	14.77
BSRI_M	85.24	18.33	84.61	18.90	4.27	0.92	4.19	0.95
BSRI_F	87.58	14.67	91.12	15.63	4.49	0.73	4.43	0.85
BSRI_N	85.92	12.77	86.36	12.19	4.31	0.58	4.30	0.72

*^1^transgressive theme; ^2^emotional and romantic themes; ^3^dominance/submission; ^4^variety of partners; ^5^explicit sexuality.*

**FIGURE 1:**
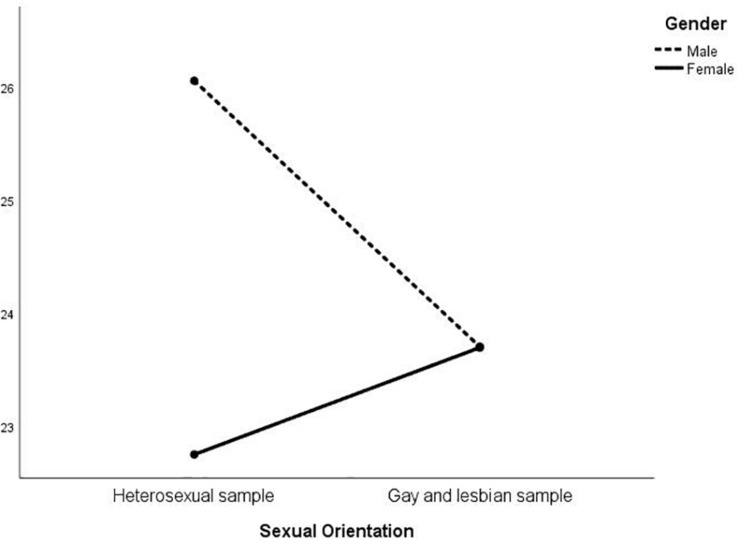
Interaction of gender × sexual orientation on EIQ F1-Trasgressive themes.

**FIGURE 2:**
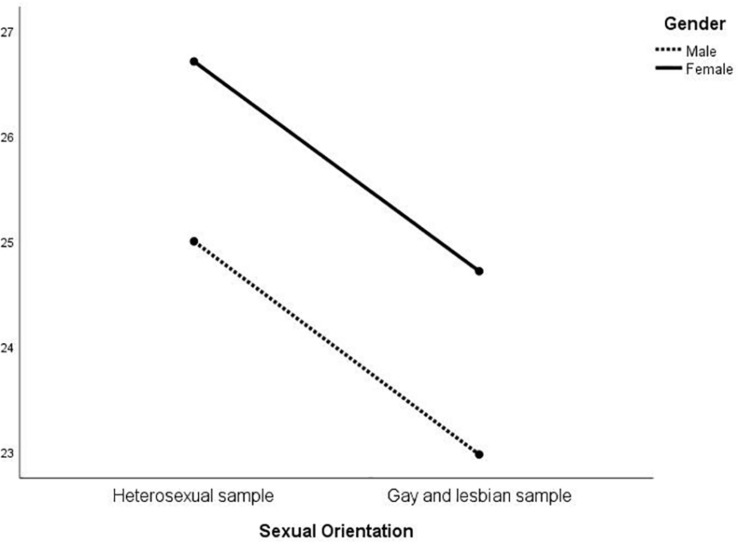
Interaction of gender × sexual orientation on EIQ F2-Emotional and romantic themes.

**FIGURE 3:**
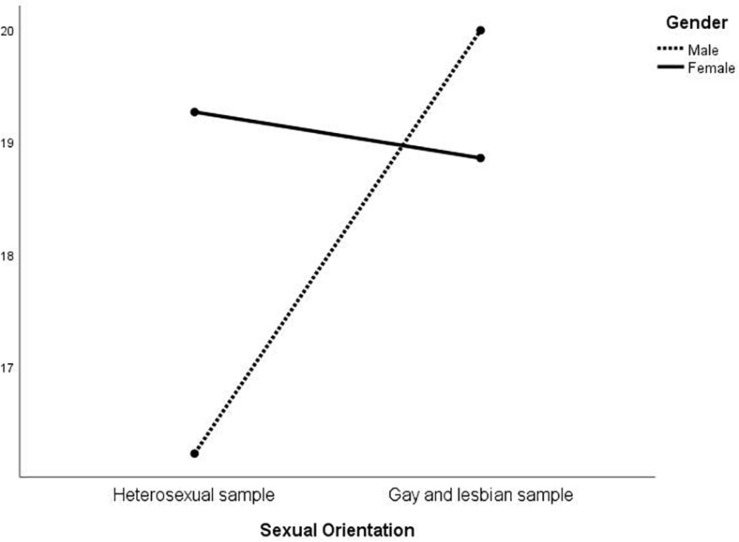
Interaction of gender × sexual orientation on EIQ F3-dominance/submission.

**FIGURE 4:**
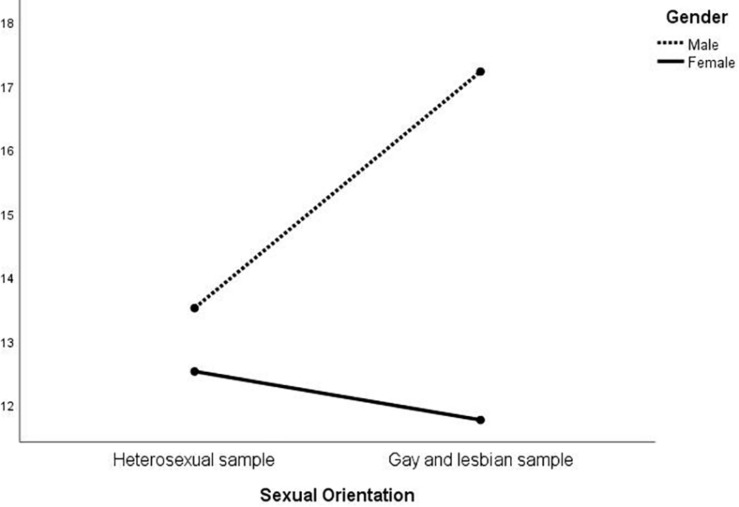
Interaction of gender × sexual orientation on EIQ F4-Variety of partners.

**FIGURE 5:**
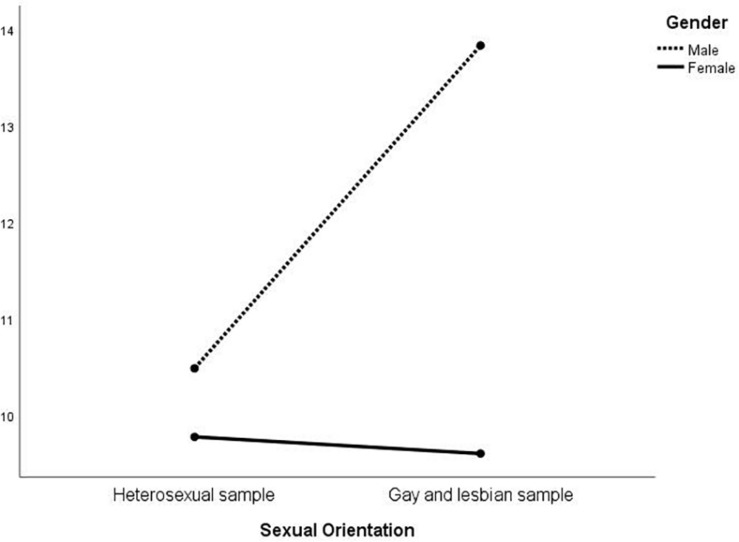
Interaction of gender × sexual orientation on EIQ F5. Explicit sexuality.

MANOVA was then conducted on the EIQ factors to investigate whether an interaction between gender and sexual orientation was present (Table 3). At the multivariate level, there were significant differences in gender [*F*_(_1_, _574_)_ = 31.01; *p* < 0.01], sexual orientation [*F*_(_1_, _574_)_ = 15.36; *p* < 0.01], and in the interaction between the latter two [*F*_(_1_, _574_)_ = 24.11; *p* < 0.01]. At the univariate level, sexual orientation was significant for all the five factors except for F1; gender was always significant, except for F3; the interaction was significant for all but F2. The results are represented in Figures 1–5. Specifically, on F1, men scored higher than women only in the heterosexual group [*F*_(_1_, _574_)_ = 6.23; *p* < 0.01]; on F2 (Figure 2), women scored higher than men regardless of their sexual orientation [*F*_(_1_, _574_)_ = 10.98; *p* < 0.01], but the heterosexual group scored higher than the gay men and lesbian women [*F*_(_1_, _574_)_ = 14.89; *p* < 0.01]; on F3, non-heterosexual individuals scored higher than heterosexuals [*F*_(_1_, _574_)_ = 8.55; *p* < 0.01] and gender difference was significant only for the heterosexual group, with women scoring higher than men (Figure 3); on F4 (Figure 4), men showed significantly higher scores than women [*F*_(_1_, _574_)_ = 66.71; *p* < 0.01] and gay and lesbian individuals scored significantly higher than heterosexuals [*F*_(_1_, _574_)_ = 13.91; *p* < 0.01]; finally, on F5 (Figure 5) men scored higher than women [*F*_(_1_, _574_)_ = 50.81; *p* < 0.01] and gay men and lesbian women higher than heterosexuals [*F*_(_1_, _574_)_ = 20.98; *p* < 0.01]. For both F4 and F5, gender differences were stronger in the gay men and lesbian women group.

**TABLE 3:** Between-subject effects on the EIQ factors.

		**df**	***F***	***p***
Sexual orientation	F1^1^	1	1.12	0.29
	F2^2^	1	14.89	< 0.001
	F3^3^	1	8.55	< 0.001
	F4^4^	1	13.9	< 0.001
	F5^5^	1	20.98	< 0.001
Gender	F1^1^	1	6.23	< 0.05
	F2^2^	1	10.98	< 0.001
	F3^3^	1	2.74	0.09
	F4^4^	1	66.71	< 0.001
	F5^5^	1	50.81	< 0.001
Sexual orientation × gender	F1^1^	1	6.16	< 0.01
	F2^2^	1	0.01	0.97
	F3^3^	1	13.23	< 0.001
	F4^4^	1	31.96	< 0.001
	F5^5^	1	25.8	< 0.001

*^1^transgressive theme; ^2^emotional and romantic themes; ^3^dominance/submission; ^4^variety of partners; ^5^explicit sexuality.*

For the IRQ and for SPQ, ANOVA (Table 4) and MANOVA (Tables 5, 6) were, respectively, conducted for the same purpose. In the IRQ, gay men and lesbian women scored significantly higher than heterosexuals, regardless of the gender, while, on the SPQ, the effect of the interaction between gender and sexual orientation was statistically significant for both the feminine [*F*_(_2_, _574_)_ = 4.68; *p* < 0.03] and the masculine [*F*_(_2_, _574_)_ = 4.91; *p* < 0.02] subscales; the effect of gender was significant only on the feminine subscale [*F*_(_2_, _574_)_ = 4,44; *p* < 0.04], with women scoring higher than men only in the heterosexual group.

**TABLE 4:** Between-subject effects of gender and sexual orientation on the IRQ.

	**df**	***F***	***p***
Gender	1	0.03	0.86
Sexual orientation	1	6.65	<0.01
Gender × sexual orientation	1	0.92	0.34

**TABLE 5:** MANOVA multivariate test of the effects on the SPQ.

**Effect**	***F***	**df**	**Error df**	***p***
Gender	3.74	2	542	<0.05
Sexual orientation	1.87	2	542	0.16
Gender × sexual orientation	2.76	2	542	0.06

**TABLE 6:** Between-subject effects on the feminine and masculine subscales of the SPQ.

		**df**	***F***	***p***
Gender	SPQ_M	1	0.13	0.71
	SPQ_F	1	4.44	<0.05
Sexual orientation	SPQ_M	1	1.51	0.22
	SPQ_F	1	3.67	0.06
Gender × sexual orientation	SPQ_M	1	4.91	<0.05
	SPQ_F	1	4.68	<0.05

MANOVA was also used to explore the main effects on the BSRI: at the multivariate level, only gender was significant [*F*_(_1_, _574_)_ = 3.06; *p* < 0.028]; at the univariate level, gender was significant only for the feminine subscale [*F*_(_1_, _574_)_ = 6.06; *p* < 0.01]. According to the BSRI, 65.8% of heterosexual men, 59.8% of heterosexual women, 80.9% of gay men, and 72.6% of lesbian women had neutral scores.

## Discussion

In the present paper, we wanted to analyze differences and the relationship between sexual fantasies and gender roles in heterosexual and lesbian and gay populations. We first hypothesized that heterosexuals will show more normative sexual fantasies than gay and lesbian participants (Hypothesis 1) (Bhugra et al., 2006) and that a change toward androgynous characteristics is expected in women (both heterosexual and lesbian women) (Diekman and Eagly, 2000; Zafra and Garcia-Retamero, 2011), in order to adhere to feminine social expectations (Hypothesis 2).

According to our results, Hypothesis 1 was verified only in the female sample. Indeed, lesbian women scored slightly higher than heterosexual women on transgressive sexual fantasies and lower on emotional-romantic ones, which may, however, be a consequence of other aspects such as openness about sexuality, past sexual experiences, and relationship status (Peterson et al., 2010; Vrangalova and Savin-Williams, 2010). The general findings are in accordance with the hypothesis that women have a preference for emotive-romantic contents, while men tend to report more explicit sexual fantasies (F5) as well as a variety of partners (F4) (Singer, 1985; Zurbriggen and Yost, 2004). Nevertheless, gender differences on F4 and F5 were more prominent between the gay men and lesbian women groups, and heterosexual men reported engaging in emotional-romantic sexual fantasies more often than gay men. Thus, the idea that men maintain the same pattern of preferences regardless of sexual orientation (Masters et al., 1982) does not totally apply to the current sample. These results, which are not in line with the existing literature on sexual fantasies and sexual orientation, may be a consequence of the social pressure that gay men may feel to appear more masculine: romantic sexual fantasies can be perceived as a “feminine type” of erotic thought (Hunt et al., 2015; Salvati et al., 2019) and may thus be rejected by gay men.

Subsequently, Hypothesis 2 was also only partially verified. Most of the sample showed androgynous characteristics. This data is in line with the evolutionary idea that androgynous people are more flexible to environmental tasks and that acquiring androgynous roles could be an adaptive pattern developed after evolutive pressures (Green and Kenrick, 1994; Woodhill and Samuels, 2004; Cobb et al., 2009). Heterosexual women, but not lesbian women, showed a pattern of high social pressure to conform to feminine expectations together with lower scores in the IRQ. The same was true for gay men but not for heterosexual men. Moreover, a moderate positive correlation was present between the feminine subscale of the SPQ and the IRQ. Interestingly, regardless of gender and sexual orientation and the slightly but significative differences among the groups, individuals scored above the average on the IRQ and also on both subscales of the SPQ. It is speculated that differentiating between masculine and feminine traits and behaviors may be outdated, at least in the young Italian population: individuals might be expected to behave in a more sensitive and caring manner in certain circumstances, such as in interpersonal relationships, and at the same time to adopt more masculine behaviors in others, such as in the workplace, regardless of their biological sex and sexual orientation. Indeed, our sample showed an internalized pattern of androgynous characteristics, which might be representative of a general adaptation to deal with the demands and expectations of modern Italian society. Notably, a strong positive correlation was found between the two subscales of the SPQ, which could be viewed as a further indicator of the androgynous nature of the experienced social pressure.

## Conclusion

Our findings suggest that sexual fantasies and gender roles are relatively separate concepts that have little influence on one another. Indeed, despite the shift toward androgynous characteristics, the traditional themes of sexual fantasies associated with men and women appear to remain relatively unaffected in the heterosexual population, although differences are less prominent than was initially proposed. In fact, the extent of gender differences in sexual fantasies may be rather linked to internalized sexual stereotypes that are not affected by social variations of the roles attributed to men and women. The aim of the present research was to investigate how gender and sexual orientation influence sexual fantasies and gender roles as well as examining whether traditional theories about sexual fantasies and gender roles could be applied to Italian heterosexual and gay men and lesbian women. The themes of sexual fantasies typically associated with men and women appeared relatively unchanged, despite a general shift toward androgynous traits and behaviors and irrespective of sexual orientation.

### Limitations

One main limitation of the present study is that no control for social desirability was included in the battery. Additionally, our study is based on cross-sectional data registered through self-report measures: longitudinal studies are needed to understand the relationships between the study variables better, using other measurements such as structured interviews. Moreover, we used *ad hoc* questionnaires, specifically developed for the present research, that require future studies to be validated in the general population. Another major limitation is denoted by the restrictive exclusion criteria, which limit not only the generalizability of the results but also their applicability in the study of individual differences. It is worth noting that no information could be collected from individuals who did not voluntarily participate in the research. The study of the differences between those who are willing to participate in research about sexuality and those who are not would be central to the study of sexuality in general (Zurbriggen and Yost, 2004), as it can provide insights that may be particularly relevant in clinical contexts. Future research should focus on assessing sexual fantasies in non-binary individuals, whose perspective on gender may provide valuable insights on how sexual fantasies and gender roles are related. Furthermore, comparing individuals who endorse exclusively stereotypical behaviors with those who do the opposite could expand our knowledge about gender discrepancy and its consequences on the person.

### Future Directions

Future research is needed to expand the present findings to the general population and to deepen our knowledge about gender discrepancies and their consequences for the individual. Future research should also consider how sexual fantasies vary with age and experiences. A longitudinal study would also be particularly effective in determining whether possible differences are due to aging or rather to different patterns of social pressure, culture, and sex guilt. Finally, studies assessing the relationship between gender roles, sexual orientation, gender differences, and sexual fantasies should also focus on non-binary, intersexual, and transgender populations in order to develop inclusive and supportive intervention programs.

## Data Availability Statement

The datasets generated for this study are available on request to the corresponding author.

## Ethics Statement

The research was conducted in accordance with the ethical principles stated in the Declaration of Helsinki (2013). The patients/participants provided their written informed consent to participate in this study.

## Author Contributions

All authors listed have made a substantial, direct and intellectual contribution to the work, and approved it for publication.

## Conflict of Interest

The authors declare that the research was conducted in the absence of any commercial or financial relationships that could be construed as a potential conflict of interest.

The reviewer MS declared a shared affiliation, with no collaboration, with one of the authors FN to the handling Editor at the time of review.

## References

[B1] Allen L. (2003). Girls want sex, boys want love: resisting dominant discourses of (Hetero) sexuality. *Sexualities* 6 215–236. 10.1177/1363460703006002004

[B2] American Psychiatric Association, (2013). *Diagnostic And Statistical Manual Of Mental Disorders*, 5th Edn Washington, DC: Author.

[B3] Bandura A. (1986). *Social Foundations of Thought and Action: A Social Cognitive Theory. Social Foundations of Thought and Action: A Social Cognitive Theory.* Englewood Cliffs, NJ: Prentice-Hall, Inc.

[B4] Baumeister R. F. Catanese K. R. Vohs K. D. (2001). Is there a gender difference in strength of sex drive? Theoretical views, conceptual distinctions, and a review of relevant evidence. *Personal. Soc. Psychol. Rev.* 5 242–273. 10.1207/S15327957PSPR0503_5

[B5] Bem S. L. (1974). The measurement of psychological androgyny. *J. Consult. Clin. Psychol.* 42 155–162. 10.1037/h0036215 4823550

[B6] Bem S. L. (1993). *The Lenses of Gender: Transforming the Debate on Sexual Inequality. The Lenses of Gender: Transforming the Debate on Sexual Inequality.* New Haven, CT: Yale University Press.

[B7] Bhugra D. Rahman Q. Bhintade R. (2006). Sexual fantasy in gay men in India: a comparison with heterosexual men. *Sex. Relationsh. Ther.* 21 197–207. 10.1080/14681990600554207

[B8] Birnbaum G. E. Simpson J. A. Weisberg Y. J. Barnea E. Assulin-Simhon Z. (2012). Is it my overactive imagination? The effects of contextually activated attachment insecurity on sexual fantasies. *J. Soc. Pers. Relationsh.* 29 1131–1152. 10.1177/0265407512452978

[B9] Blashill A. J. Powlishta K. K. (2009). Gay stereotypes: the use of sexual orientation as a cue for gender-related attributes. *Sex Roles A J. Res.* 61 783–793. 10.1007/s11199-009-9684-7

[B10] Bosak J. Eagly A. Diekman A. Sczesny S. (2017). Women and men of the past, present, and future: evidence of dynamic gender stereotypes in Ghana. *J. Cross Cult. Psychol.* 49 115–129. 10.1177/0022022117738750

[B11] Brown J. J. Hart D. H. (1977). Correlates of females’ sexual fantasies. *Percept. Mot. Skills* 3(Pt 1), 819–825. 10.2466/pms.1977.45.3.819 600641

[B12] Cado S. Leitenberg H. (1990). Guilt reactions to sexual fantasies during intercourse. *Arch. Sex. Behav.* 19 49–63. 10.1007/BF01541825 2327895

[B13] Carpenter D. Janssen E. Graham C. Vorst H. (2008). Women’s scores on the Sexual Inhibition/Sexual Excitation Scales (SIS/SES): gender similarities and differences. *J. Sex Res.* 45 36–48. 10.1080/00224490701808076 18321029

[B14] Cobb R. A. Walsh C. E. Priest J. B. (2009). The cognitive-active gender role identification continuum. *J. Fem. Fam. Ther.* 21 77–97. 10.1080/08952830902911339

[B15] Constantin A. Voicu M. (2014). Attitudes towards gender roles in cross-cultural surveys: content validity and cross-cultural measurement invariance. *Soc. Indic. Res.* 123 733–751. 10.1007/s11205-014-0758-8

[B16] Cox W. T. L. Devine P. G. Bischmann A. A. Hyde J. S. (2016). inferences about sexual orientation: the roles of stereotypes. faces, and the gaydar myth. *J. Sex Res.* 53 157–171. 10.1080/00224499.2015.1015714 26219212 PMC4731319

[B17] Critelli J. W. Bivona J. M. (2008). Women’s erotic rape fantasies: an evaluation of theory and research. *J. Sex Res.* 45 57–70. 10.1080/00224490701808191 18321031

[B18] Dean M. L. Tate C. C. (2017). Extending the legacy of sandra bem: psychological androgyny as a touchstone conceptual advance for the study of gender in psychological science. *Sex Roles* 76 643–654. 10.1007/s11199-016-0713-z

[B19] Diekman A. B. Eagly A. H. (2000). Stereotypes as dynamic constructs: women and men of the past, present, and future. *Personal. Soc. Psychol. Bull.* 26 1171–1188. 10.1177/0146167200262001 30761034

[B20] Diekman A. B. Johnston A. M. Loescher A. L. (2013). Something old, something new: evidence of self-accommodation to gendered social change. *Sex Roles A J. Res.* 68 550–561. 10.1007/s11199-013-0263-6

[B21] D’Urso G. Pace U. (2019). Homophobic bullying among adolescents: the role of insecure-dismissing attachment and peer support. *J. LGBT Youth* 16 173–191. 10.1080/19361653.2018.1552225

[B22] D’Urso G. Petruccelli I. Grilli S. Pace U. (2019). Risk factors related to cognitive distortions toward women and moral disengagement: a study on sex offenders. *Sexual. Cult.* 23 544–557. 10.1007/s12119-018-9572-9

[B23] Eagly A. H. (1987). *Sex Differences in Social Behavior: A Social-Role Interpretation. Sex Differences in Social Behavior: A Social-Role Interpretation.* New Jerrsy, NJ: Lawrence Erlbaum Associates, Inc.

[B24] Fausto-Sterling A. (2012). The dynamic development of gender variability. *J. Homosexual.* 59 398–421. 10.1080/00918369.2012.653310 22455327

[B25] Fontanesi L. Renaud P. (2014). Sexual presence: toward a model inspired by evolutionary psychology. *New Ideas Psychol.* 33 1–7. 10.1016/j.newideapsych.2013.10.001

[B26] Geis F. L. (1993). *Self-Fulfilling Prophecies: A Social Psychological View of Gender. In The Psychology of Gender.* New York, NY: Guilford Press, 9–54.

[B27] Gelman S. A. Taylor M. G. Nguyen S. P. Leaper C. Bigler R. S. (2004). Mother-child conversations about gender: understanding the acquisition of essentialist beliefs. *Monogr. Soc. Res. Child Dev.* 69 1–14. 10.1111/j.1540-5834.2004.06901002.x15566544

[B28] Goldey K. L. Avery L. R. van Anders S. M. (2014). Sexual fantasies and Gender/Sex: a multimethod approach with quantitative content analysis and hormonal responses. *J. Sex Res.* 51 917–931. 10.1080/00224499.2013.798611 23998565

[B29] Green B. L. Kenrick D. T. (1994). The attractiveness of gender-typed traits at different relationship levels: androgynous characteristics may be desirable after all. *Personal. Soc. Psychol. Bull.* 20 244–253. 10.1177/0146167294203002

[B30] Hall N. M. Pichon L. C. (2014). Gender roles, sociosexuality, and sexual behavior among US Black women. *Health Psychol. Behav. Med.* 2 171–182. 10.1080/21642850.2014.882236 25614852 PMC4299751

[B31] Heine S. J. Lehman D. R. Markus H. R. Kitayama S. (1999). *Psychol. Rev.* 106 766–794. 10.1037/0033-295X.106.4.766 10560328

[B32] Higgins E. T. (1987). Self-discrepancy: a theory relating self and affect. *Psychol. Rev. Am. Psychol. Assoc.* 3 319–340. 10.1037/0033-295X.94.3.319 3615707

[B33] Higgins L. T. Zheng M. Liu Y. Sun C. H. (2002). Attitudes to marriage and sexual behaviors: a survey of gender and culture differences in China and United Kingdom. *Sex Roles* 46 75–89. 10.1023/A:1016565426011

[B34] Hoffman R. M. Borders L. D. (2001). Twenty-five years after the Bem Sex-Role Inventory: a reassessment and new issues regarding classification variability. *Meas. Eval. Couns. Dev.* 34 39–55. 10.1080/07481756.2001.12069021

[B35] Hunt C. Fasoli F. Carnaghi A. Cadinu M. (2015). Masculine self-presentation and distancing from femininity in gay men: an experimental examination of the role of masculinity threat. *Psychol. Men Masc.* 17 108–112. 10.1037/a0039545

[B36] IBM, (2012). *IBM SPSS Advanced Statistics 23.* Armonk, NY: IBM Corp, 10.1080/02331889108802322

[B37] Kabbash I. A. Zidan O. O. Salem S. M. (2019). Perceptions of gender roles in sexual relations and the sexual experiences of medical students in the Nile Delta of Egypt. *Sex. Cult.* 23 310–324. 10.1007/s12119-018-9559-6

[B38] Kinsey’s A. C. Pomeroy W. B. Martin C. E. (1948). *Sexual Behavior in the Human Male.* Philadelphia: Saunders Philadelphia.

[B39] Kite M. E. Deaux K. Haines E. L. (2008). *Gender Stereotypes. In Psychology of Women: A Handbook of Issues and Theories*, 2nd Edn Westport, CT: Greenwood Publishing Group, 205–236.

[B40] Knafo D. Jaffe Y. (1984). Sexual fantasizing in males and females. *J. Res. Personal.* 18 451–462. 10.1016/0092-6566(84)90004-7

[B41] Koch S. C. Luft R. Kruse L. (2005). Women and leadership – 20 years later: a semantic connotation study. *Soc. Sci. Inf.* 44 9–39. 10.1177/0539018405050433

[B42] Lehavot K. Lambert A. J. (2007). Toward a greater understanding of antigay prejudice: on the role of sexual orientation and gender role violation. *Basic Appl. Soc. Psychol.* 29 279–292. 10.1080/01973530701503390

[B43] Leitenberg H. Henning K. (1995). Sexual fantasy. *Psychol. Bull.* 117 469–496. 7777650

[B44] Lips H. M. (2017). Sandra Bem: naming the impact of gendered categories and identities. *Sex Roles* 76 627–632. 10.1007/s11199-016-0664-4

[B45] Littlefield M. B. (2004). Gender role identity and stress in african american women. *J. Hum. Behav. Soc. Environ.* 8 93–104. 10.1300/J137v08n04_06

[B46] Martin C. L. Fabes R. A. (2001). The stability and consequences of young children’s same-sex peer interactions. *Dev. Psychol.* 37 431–446. 10.1037/0012-1649.37.3.431 11370917

[B47] Martin C. L. Ruble D. N. (2010). Patterns of gender development. *Ann. Rev. Psychol.* 61 353–381. 10.1146/annurev.psych.093008.100511 19575615 PMC3747736

[B48] Masters W. H. Johnson V. E. översättning S. Lisper H.-O. (1982). Homosexuality in perspective. *Scand. J. Behav. Ther.* 11 54–56. 10.1080/16506078209456227

[B49] Moyano N. Sierra J. C. (2013). Relationships between personality traits and positive/negative sexual cognitions. *Int. J. Clin. Health Psychol.* 13 189–196. 10.1016/s1697-2600(13)70023-1

[B50] Nathanson A. I. Wilson B. J. McGee J. Sebastian M. (2006). Counteracting the effects of female stereotypes on television via active mediation. *J. Commun.* 52 922–937. 10.1111/j.1460-2466.2002.tb02581.x

[B51] Nguyen A. B. Clark T. T. Hood K. B. Corneille M. A. Fitzgerald A. Y. Belgrave F. Z. (2010). Beyond traditional gender roles and identity: does reconceptualisation better predict condom-related outcomes for African-American women? *Cult. Health Sex.* 12 603–617. 10.1080/13691051003658127 20234960 PMC6367702

[B52] Nobre P. Gouveia J. P. Gomes F. A. (2003). sexual dysfunctional beliefs questionnaire: an instrument to assess sexual dysfunctional beliefs as vulnerability factors to sexual problems. *Sex. Relationsh. Ther.* 18 171–204. 10.1080/1468199031000061281

[B53] Okazaki S. (2002). Influences of culture on Asian Americans’ sexuality. *J. Sex Res.* 39 34–41. 10.1080/00224490209552117 12476254

[B54] Panzeri M. Fontanesi L. (2014). “Factors affecting women’s sexual arousal: a focus group study in italy,” in *Proceedings of the 16th Annual Congress of the European Society for Sexual Medicine and the 12th Congress of the European Sexology Federation.* Istanbul, 94–108.

[B55] Panzeri M. Fontanesi L. Gardin E. (2015). L’Erotic Imagery Questionnaire (EIQ). Una prima valutazione psicometrica. *Riv. Di Sessuologia Clin.* 39 112–127.

[B56] Pelletier L. A. Herold E. S. (1988). The relationship of age, sex guilt, and sexual experience with female sexual fantasies. *J. Sex Res.* 24 250–256. 10.1080/00224498809551420 22375657

[B57] Peterson Z. D. Janssen E. Laan E. (2010). Women’s sexual responses to heterosexual and lesbian erotica: the role of stimulus intensity, affective reaction, and sexual history. *Arch. Sex. Behav.* 39 880–897. 10.1007/s10508-009-9546-y 19856092

[B58] Pietropinto A. Simenauer J. (1977). *Beyond the Male Myth.* New York: Times Books.

[B59] Price J. H. Allensworth D. D. Hillman K. S. (1985). Comparison of sexual fantasies of homosexuals and of heterosexuals. *Psycholo. Rep.* 57 871–877. 10.2466/pr0.1985.57.3.871 4080916

[B60] Price J. H. Miller P. A. (1984). Sexual fantasies of black and of white college students. *Psychol. Rep.* 54 1007–1014. 10.2466/pr0.1984.54.3.1007

[B61] Renaud C. A. Byers E. S. (2001). Positive and negative sexual cognitions: subjective experience and relationships to sexual adjustment. *J. Sex Res.* 38 252–262. 10.1080/00224490109552094

[B62] Ross L. (1977). “The intuitive psychologist and his shortcomings: distortions in the attribution process,” in *Advances in Experimental Social Psychology*, Vol. 10 ed. Berkowitz E. S. P., (Cambridge, MA: Academic Press), 173–220. 10.1016/s0065-2601(08)60357-3

[B63] Rudman L. A. Phelan J. E. (2010). The effect of priming gender roles on women’s implicit gender beliefs and career aspirations. *Soc. Psychol.* 41 192–202. 10.1027/1864-9335/a000027

[B64] Salvati M. Pistella J. Ioverno S. Giacomantonio M. Baiocco R. (2017). Attitude of italian gay men and italian lesbian women towards gay and Lesbian gender-typed scenarios. *Sex. Res. Soc. Policy J. NSRC* 15 312–328. 10.1007/s13178-017-0296-7

[B65] Salvati M. Piumatti G. Giacomantonio M. Baiocco R. (2019). Gender stereotypes and contact with gay men and lesbians: the mediational role of sexism and homonegativity. *J. Commun. Appl. Soc. Psychol.* 29 461–473. 10.1002/casp.2412

[B66] Shanor K. (1977). *The Fantasy Files: A Study of the Sexual Fantasies of Contemporary Women.* New York, NY: Dial Press.

[B67] Singer B. (1985). A comparison of evolutionary and environmental theories of erotic response part II: empirical arenas. *J. Sex Res.* 21 345–374. 10.1080/00224498509551275

[B68] Sirin S. R. McCreary D. R. Mahalik J. R. (2004). Differential reactions to men and women’s gender role transgressions: perceptions of social status, sexual orientation, and value dissimilarity. *J. Men’s Stud.* 12 119–132. 10.3149/jms.1202.119

[B69] Smirles K. E. (2017). Raising consciousness of gender roles through cross-cultural analysis: a course on women and leadership for Japanese women. *Psychol. Women Q.* 41 389–392. 10.1177/0361684317701424

[B70] Starr C. R. Zurbriggen E. L. (2017). Sandra Bem’s gender schema theory after 34 years: a review of its reach and impact. *Sex Roles* 76 566–578. 10.1007/s11199-016-0591-4

[B71] Steele C. M. (1997). A threat in the air: how stereotypes shape intellectual identity and performance. *Am. Psychol.* 52 613–629. 10.1037//0003-066x.52.6.613 9174398

[B72] Sue D. (1979). Erotic fantasies of college students during coitus. *J. Sex Res.* 15 299–305. 10.1080/00224497909551053

[B73] Traeen B. Stigum H. Søorensen D. (2002). Sexual diversity in Urban Norwegians. *J. Sex Res.* 39 249–258. 10.1080/00224490209552148 12545407

[B74] Tseng W.-S. (2003). *Clinician’s Guide to Cultural Psychiatry.* San Diego: Academic Press.

[B75] Usui C. Rose S. Kageyama R. (2003). Women, institutions, and leadership in Japan. *Asian Perspect.* 27 85–123.

[B76] Vrangalova Z. Savin-Williams R. C. (2010). Correlates of same-sex sexuality in heterosexually identified young adults. *J. Sex Res.* 47 92–102. 10.1080/00224490902954307 19431038

[B77] Weinberg M. S. Lottes I. Shaver F. M. (2000). Sociocultural correlates of permissive sexual attitudes: a test of Reiss’s hypotheses about Sweden and the United States. *J. Sex Res.* 37 44–52. 10.1080/00224490009552019

[B78] Wilson G. D. (1997). Gender differences in sexual fantasy: an evolutionary analysis. *Personal. Indiv. Diff.* 22 27–31. 10.1016/S0191-8869(96)00180-8 20697937

[B79] Wolke D. Woods S. Bloomfield L. Karstadt L. (2000). The association between direct and relational bullying and behaviour problems among primary school children. *J. Child Psychol. Psychiatry Allied Discipl.* 41 989–1002. 10.1111/1469-7610.00687 11099116

[B80] Woodhill B. M. Samuels C. A. (2004). Desirable and undesirable androgyny: a prescription for the twenty-first century. *J Gen. Stud.* 13 15–28. 10.1080/09589236.2004.10599911

[B81] Wu Y. Ku L. Zaroff C. M. (2016). sexual arousal and sexual fantasy: the influence of gender, and the measurement of antecedents and emotional consequences in Macau and the United States. *Int. J. Sex. Health* 28 55–69. 10.1080/19317611.2015.1111281

[B82] Yamamoto M. Ran W. (2014). Should men work outside and women stay home? revisiting the cultivation of gender-role attitudes in Japan. *Mass Commun. Soc.* 17 920–942. 10.1080/15205436.2013.860989

[B83] Yost M. R. Zurbriggen E. L. (2006). Gender differences in the enactment of sociosexuality: an examination of implicit social motives, sexual fantasies, coercive sexual attitudes, and aggressive sexual behavior. *J. Sex Res.* 43 163–173. 10.1080/00224490609552311 16817063

[B84] Zafra E. L. Garcia-Retamero R. (2011). The impact of nontraditionalism on the malleability of gender stereotypes in Spain and Germany. *Int. J. Psychol.* 46 249–258. 10.1080/00207594.2010.551123 22044269

[B85] Zurbriggen E. L. Yost M. R. (2004). Power, desire, and pleasure in sexual fantasies. *J. Sex Res.* 41 288–300. 10.1080/00224490409552236 15497057

